# Which Clinicopathologic Parameters Suggest Primary Resistance to Palbociclib in Combination With Letrozole as the First-Line Treatment for Hormone Receptor-Positive, HER2-Negative Advanced Breast Cancer?

**DOI:** 10.3389/fonc.2021.759150

**Published:** 2021-10-21

**Authors:** Ji-Yeon Kim, Jung Min Oh, Yeon Hee Park, Jin Seok Ahn, Young-Hyuck Im

**Affiliations:** ^1^Division of Hematology-Oncology, Department of Medicine, Samsung Medical Center, Sungkyunkwan University School of Medicine, Seoul, South Korea; ^2^Biomedical Research Institute, Samsung Medical Center, Sungkyunkwan University School of Medicine, Seoul, South Korea

**Keywords:** metastatic breast cancer (MBC), hormone receptor positive (HR+), first line, CDK4/6 inhibitor, primary resistance

## Abstract

In this study, we evaluated clinical parameters to predict the primary resistance of palbociclib in combination with endocrine therapy as the first-line treatment in patients with hormone receptor (HR)+, human epidermal growth factor receptor 2 (HER2)- metastatic breast cancer (MBC). We performed a data analysis of patients diagnosed with HR+, HER2-MBC who received palbociclib plus letrozole as the first-line treatment in the metastatic setting from the clinical data warehouse in Samsung Medical Center. In this study, 305 patients were included in the final data analysis. The median follow-up duration was 31 months, and we observed 123 cases of disease progression. The median progression-free survival (PFS) was 28.7 months, and 38 patients (12.5%) had less than a 6-month PFS. The multivariate analysis suggested that primary resistance to adjuvant endocrine therapy (ET) (hazard ratio: 1.91), presence of liver metastasis (hazard ratio: 2.17), initial elevation of serum CA-15-3 (hazard ratio: 1.99), weak positivity of estrogen receptor (ER) (hazard ratio: 2.28), Ki-67 3+ or 4+ (hazard ratios: 2.58 and 10.28), and presence of mutation (hazard ratio: 9.59) were associated with a short PFS duration. A further prediction model was developed with data from 256 patients and 33 cases of disease progression in 6 months. This model included five factors—primary resistance to adjuvant ET (odds ratio, OR: 1.14), liver metastasis (OR: 1.56), initial CA-15-3 elevation (OR: 1.51), weak ER expression (OR: 2.22), and BRCA2 mutation (OR: 2.85)—and the area under the receiver operating characteristic curve was 0.842 (95% CI: 0.775, 0.909; *p* < 0.001). Finally, we divided them into four risk groups according to the prediction model with the five risk factors. These four groups had different PFS (*p* < 0.001) and primary resistance of palbociclib with letrozole [OR of group 2 *vs.* group 1 (ref): 2.18 (*p* = 0.002), OR of group 3: 3.91 (*p* < 0.001), and OR of group 4: 4.25 (*p* < 0.001)]. We developed a prediction model of primary resistance to palbociclib with letrozole as the first-line treatment for HR+, HER2-MBC. Our prediction model might be helpful for considering the first-line treatment strategies. Further well-designed clinical trials would be warranted to validate our prediction model.

## Introduction

Hormone receptor (HR)+, human epidermal growth factor 2 (HER2)-breast cancer (BC) is the most commonly diagnosed subset of BC, accounting for 60–70% of all cases (1, 2). Endocrine therapy (ET) is the standard strategy as the initial therapy for metastatic disease in HR+, HER2-BC, even in the presence of visceral metastases, unless visceral crisis is present (3, 4).

Cyclin-dependent kinases (CDKs) play an important role in cell cycle regulation. Cyclin D1, the binding partner of CDK4/6, is often overexpressed in patients with HR+, HER2-BC, leading to the continuous activation of the cyclin D1–CDK4/6 complex (5). The interaction of cyclin D1 with CDK4/6 facilitates the hyperphosphorylation of retinoblastoma (Rb), leading to cell cycle progression through the G1 checkpoint into the S phase (6, 7). Large prospective clinical trials consistently indicate that CDK4/6 inhibitors, in combination with ET, significantly prolong the duration of progression-free survival (PFS) for HR+, HER2-metastatic BC (MBC) (8–13). Moreover, CDK4/6 inhibitors, in combination with ET, demonstrated a benefit in HR+, HER2-MBC overall survival (OS) (14, 15). The development of CDK 4/6 inhibitors has changed the paradigm of HR+, HER2-MBC management. Palbociclib, ribociclib, and abemaciclib, all orally active, highly selective, and reversible inhibitors of CDK4/6, have been approved by the Food and Drug Administration (FDA) for the treatment of HR+, HER2-MBC in combination with ET (7, 16). The current treatment guidelines suggest the combination of CDK4/6 inhibitors with ET to be the first- or second-line treatment for HR+, HER2-MBC unless visceral crisis is present (3, 4).

Despite the widespread use of CDK4/6 inhibitors in HR+, HER2-advanced BC, their efficacy with ET may be limited by the development of *de novo* or acquired resistance. Previous clinical trials showed that approximately 10% of patients with advanced HR+, HER2-BC receiving the first-line treatment consisting of CDK4/6 inhibitors with ET had less than 6 months of PFS, suggesting primary endocrine resistance (8–10). There have been many efforts to unveil the molecular mechanisms of endocrine resistance, including ESR1 mutations, RB1 mutation, overactivation of CDK 4/6, epigenetic alterations, activation of the mammalian target of rapamycin signaling pathway, inactivation of the Hippo pathway including FAT1 loss and YAP activation, and the alterations of somatic genes, such as PIK3KA, FGFR1, and AKT1 (17–23). At present, there are no known biomarkers for primary resistance in patients with HR+, HER2-advanced BC who undergo CDK 4/6 inhibitors with ET as the first-line treatment. Therefore, a new strategy to evaluate the primary resistance of CDK 4/6 inhibitors with ET as the first-line treatment was needed.

In this study, we aimed to evaluate the clinical parameters to predict the primary resistance of the first-line treatment consisting of palbociclib in combination with ET in patients with HR+, HER2-MBC.

## Patients and Methods

### Patients

We performed a data analysis of anonymized electronic medical records from the clinical data warehouse (CDW) in Samsung Medical Center (SMC). First, we extracted the data of patients who were treated with palbociclib plus aromatase inhibitor from the CDW, and then we excluded the patients who were not treated as the first-line treatment. Finally, data of patients diagnosed with HR+, HER2-MBC who received palbociclib plus an aromatase inhibitor (AI) as the first-line treatment in the metastatic setting at SMC were analyzed. The diagnostic studies for MBC included chest and abdomino-pelvic computed tomography (CT), bone scan or positron emission tomography–CT, and brain magnetic resonance imaging, if indicated, as well as histologic and immunohistochemical (IHC) examinations when the disease recurred during or after adjuvant ET or *de novo*. This study was reviewed and approved by the Institutional Review Board (IRB) of Samsung Medical Center, Seoul, South Korea (IRB no. 2021-07-131). This study was performed in accordance with the Declaration of Helsinki and the Good Clinical Practice guidelines. The requirement for informed consent was waived due to the use of de-identified medical records with clinical data.

### Breast Cancer Pathology

Histologic evaluation with hematoxylin and eosin (H&E) staining and estrogen receptor (ER), progesterone receptor (PgR), and HER2 statuses by IHC staining of MBC were assessed by at least two experienced pathologists. ER and PgR positivity were defined as Allred scores in the range of 3–8 according to IHC staining with anti-ER (Immunotech, France) and anti-PgR (Novocastra, UK) antibodies, respectively. HER2 status was evaluated using the appropriate antibody staining (DAKO, CA) or silver *in situ* hybridization (SISH). HER2 grades of 0 and 1 were defined as negative results, while grade 3 was identified as a positive result. HER2 amplification was confirmed by SISH results of 2+. In terms of Ki-67, the pathologists performed their assessment by IHC on the Ventana Discovery autostainer using the MIB-1 antibody as previously described; (24). We divided the histologic data into four groups based on the level of Ki-67 expression for further analysis: 1+ (0–25%), 2+ (25–50%), 3+ (50–75%), and 4+ (75–100%). Histologic grade and nuclear grade were also evaluated by Bloom–Richardson grading and the World Health Organization grading system, respectively (25).

### Statistical Analysis

PFS was defined as the elapsed time from the first day of palbociclib with letrozole treatment as the first-line treatment for metastatic setting to the detection of disease progression. OS was defined as the duration between the first day of palbociclib with letrozole treatment and death. PFS and OS were analyzed using the Kaplan–Meier method. Cox proportional hazard regression was used to estimate hazard ratios and 95% confidence intervals (CIs).

The binary logistic regression method was used for prediction model development. We used the Firth logistic regression method because events were not frequently observed in some variables. Variable weighting was performed by fitting a constant value (*α*) and coefficients (*β*) in Firth logistic regression, and we calculated the area under the curve (AUC) of the receiver operating characteristic (ROC) curve as the sum of weighted variables.


y=α+β1(factor 1)+β2(factor 2)+β3(factor 3)+⋯

For validation, internal validation was performed using bootstrap resampling datasets.

Two-tailed *p*-values <0.05 were considered statistically significant, and IBM SPSS Statistics, ver. 21 (IBM Co., Armonk, NY), was used for all statistical analyses.

## Results

### Baseline Characteristics

**Figure 1:**
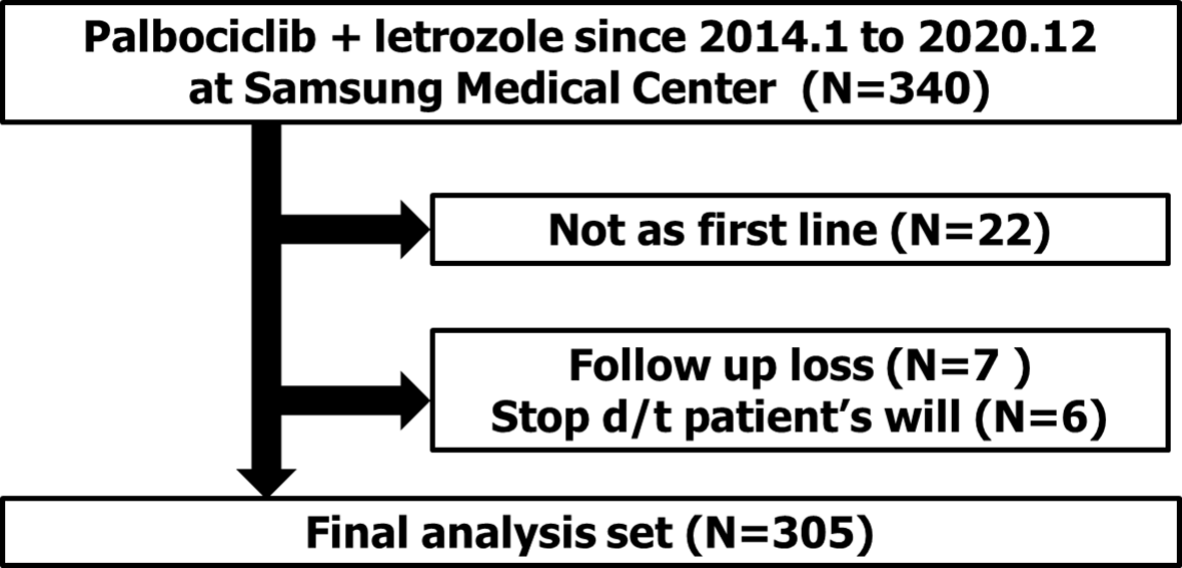
Consort diagram.

Between January 2016 and December 2020, 318 patients with MBC were treated with palbociclib and letrozole as the first-line treatment at SMC (**Figure 1**). Among all 318 patients, seven were lost to follow-up after the first cycle of palbociclib with letrozole treatment, and six patients wanted to stop the treatment without disease progression or any serious adverse events. Therefore, 305 patients were included in the final analysis.

The clinical and histologic characteristics are described in **Table 1**. The median age of the patients was 51.6 years. Of all MBC cases, 35.1% were *de novo*, and 64.9% were recurrent. In addition, 58.1% of patients with recurrent MBC had less than 12 months of being disease-free. Visceral metastases were observed in 21.3%, and three or more metastatic sites were found in 15.4%. Regarding previous treatment history, 68.7% of patients had been treated with tamoxifen with or without a GnRH agonist, and 16.7% of patients were treated with AI as adjuvant ET.

**Table 1:** Baseline patient characteristics (*N* = 305).

Characteristic	*N* (%)	Characteristic	*N* (%)
Age		Prior (neo)adjuvant chemotherapy	
Median (range)	51.6 (31.5, 86.7)	Neoadjuvant	41 (13.4)
<40 years old	23 (7.5)	Adjuvant	130 (42.6)
40–60 years old	202 (66.2)	no chemotherapy	134 (43.9)
>60 years old	80 (26.2)	Adjuvant ET (*n* = 198)	
ECOG PS		Tamoxifen	106 (53.5)
0	177 (58.0)	Tamoxifen + OFS	30 (15.2)
1	122 (40.0)	Anastrozole	14 (7.1)
≥2	5 (1.6)	Letrozole	19 (9.6)
Unknown	1 (0.4)	Unknown	10 (5.1)
Disease status at initial diagnosis		No adjuvant ET	19 (9.6)
*De novo*	107 (35.1)	Response to adjuvant ET	
Recurred	198 (64.9)	ET^-^naïve	126 (41.3)
Disease-free interval	(*n* = 198)	Primary resistance^a^	38 (12.5)
<12 months	115 (58.1)	Secondary resistance^b^	58 (19.0)
≥12 months	83 (41.9)	No resistance	83 (27.2)
Metastatic sites		ER status	
Visceral	65 (21.3)	Strong positivity^c^	278 (91.1)
Liver	60 (19.7)	Weak positivity^d^	18 (5.9)
CNS	9 (3.0)	Negative	0
Non-visceral	240 (78.7)	Unknown	9 (3.0)
Bone only	103 (33.8)	PgR status	
Stage IV LN only	29 (8.5)	Strong positivity	147 (48.2)
Number of disease sites		Weak positivity	74 (24.3)
1	158 (51.8)	Negative	75 (24.6)
2	100 (32.8)	Unknown	9 (3.0)
3 or more	47 (15.4)	Ki-67	
Germline BRCA1/2 status		1+	167 (54.8)
Not tested	244 (80.3)	2+	83 (27.2)
Tested	61 (19.7)	3+	14 (4.6)
BRCA1 mutation	0	4+	4 (1.3)
BRCA2 mutation	5 (1.6)	Unknown	37 (12.1)
No BRCA mutation	56 (18.1)		

PS, performance status; CNS, central nervous system; LN, lymph node; ET, endocrine therapy; OFS, ovarian function suppression; ER, estrogen receptor; PgR, progesterone receptor.

a Defined as breast cancer recurrence within 2 years of adjuvant ET.

b Defined as breast cancer recurrence either over 2 years since adjuvant ET or within 1 year following adjuvant ET completion.

c Allred scores 7 and 8.

d Positive with Allred scores 3–6.

With respect to histologic characteristics, strong ER positivity (defined as Allred scores of 7 and 8) was observed in 91.1% of patients, and 5.9% of patients showed weak ER positivity (defined as Allred scores of 3–6). Strong and weak PgR positivity was observed in 48.2 and 24.3% of patients, respectively, and 24.6% of patients had a negative PgR status. In terms of Ki-67, 54.8% of patients had 1+, 27.2% had 2+, 4.6% had 3+, and 1.3% had 4+.

Germline BRCA mutation testing was performed in 61 patients. In these patients, none had BRCA1 mutation, and only five patients harbored BRCA2 mutation (**Supplementary Table S1**).

### Survival Analysis of the First-Line Palbociclib With Letrozole Treatment

We performed a survival analysis of palbociclib with letrozole as the first-line treatment for HR+, HER2-MBC. In this analysis, the median PFS was 28.7 months (95% CI: 22.5, 34.9), and the median OS was not reached (**Supplementary Figure S1**). The median follow-up duration was 31 months, and we observed 123 cases of disease progression. Thirty-eight patients (12.5%) had a PFS duration of fewer than 6 months, suggesting primary resistance to palbociclib with letrozole, and 17 patients (5.6%) had less than 3 months of PFS.

### The Pathologic Characteristics Associated With the Response to Palbociclib With Letrozole

**Figure 2:**
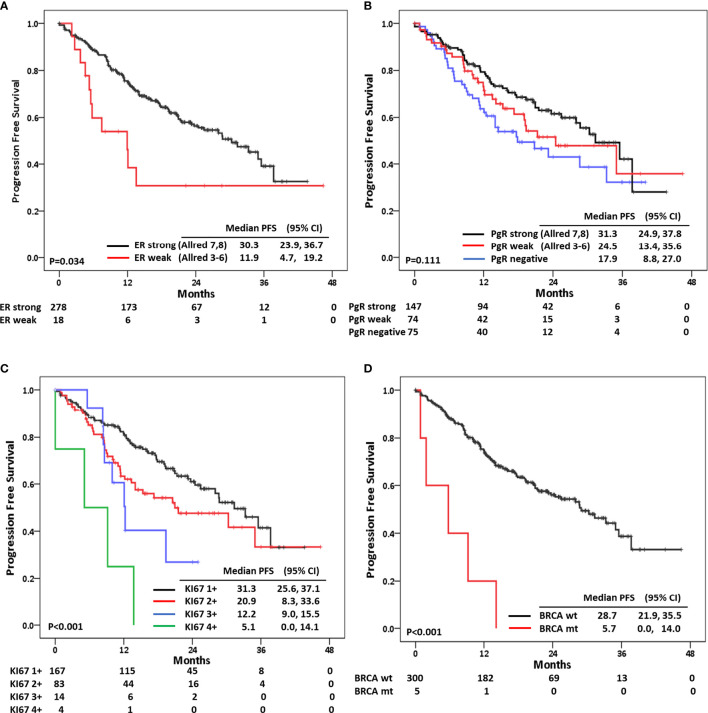
Progression-free survival according to **(A)** estrogen receptor status, **(B)** progesterone receptor status, **(C)** Ki-67 expression status, and **(D)** germline BRCA mutation.

We performed a further subset survival analysis according to baseline characteristics. First, we evaluated the impact of pathologic characteristics on PFS following palbociclib with letrozole treatment. In this analysis, BC with strong ER positivity had better PFS compared with that with weak ER positivity (median PFS, months: 30.3 *vs.* 11.9; *p* = 0.034). PgR positivity also affected PFS, and those with strong PgR positivity had a tendency to have a longer PFS compared to other groups (*p* = 0.111) (**Figures 2A, B**). Ki-67 grade also impacted PFS. Patients with Ki-67 1+ had the longest PFS among those with other grades (median PFS, months: 31.3; 95% CI: 25.6, 37.1; *p* < 0.001). In this analysis, the inverse correlation between the expression level of Ki-67 and the duration of PFS was statistically significant (**Figure 2C**).

Germline BRCA mutation also affected the efficacy of palbociclib in combination with letrozole. We found five patients harboring germline BRCA2 mutation, and they had a 5.7-month PFS compared to the 37.7-month PFS of those with BRCA wild type and the 29.0-month PFS of those without germline BRCA information (*p* < 0.001), although the number of patients with BRCA mutation was small (**Supplementary Figure S2**). For further analysis, we merged BRCA unknown and germline BRCA wild type as one category because two groups had similar PFS pattern in previous survival analysis. And we divided germline BRCA status into two categories; BRCA unknown + germline BRCA wild type and germline BRCA mutation (**Figure 2D**).

### The Clinical Characteristics Associated With Response to Palbociclib With Letrozole

In terms of clinical characteristics, the Eastern Cooperative Oncology Group (ECOG) performance status (PS) and age (under 50 *vs.* older than 50 years of age) were not associated with PFS (*p* = 0.677 for ECOG PS; *p* = 0.925 for ECOG age). *De novo* stage IV and recurred BC after curative surgery following palbociclib with letrozole treatment were also not significant in terms of PFS (*p* = 0.161). Among those with recurrent BC, the type of adjuvant endocrine therapy did not affect the efficacy of palbociclib with letrozole (*p* = 0.215).

**Figure 3:**
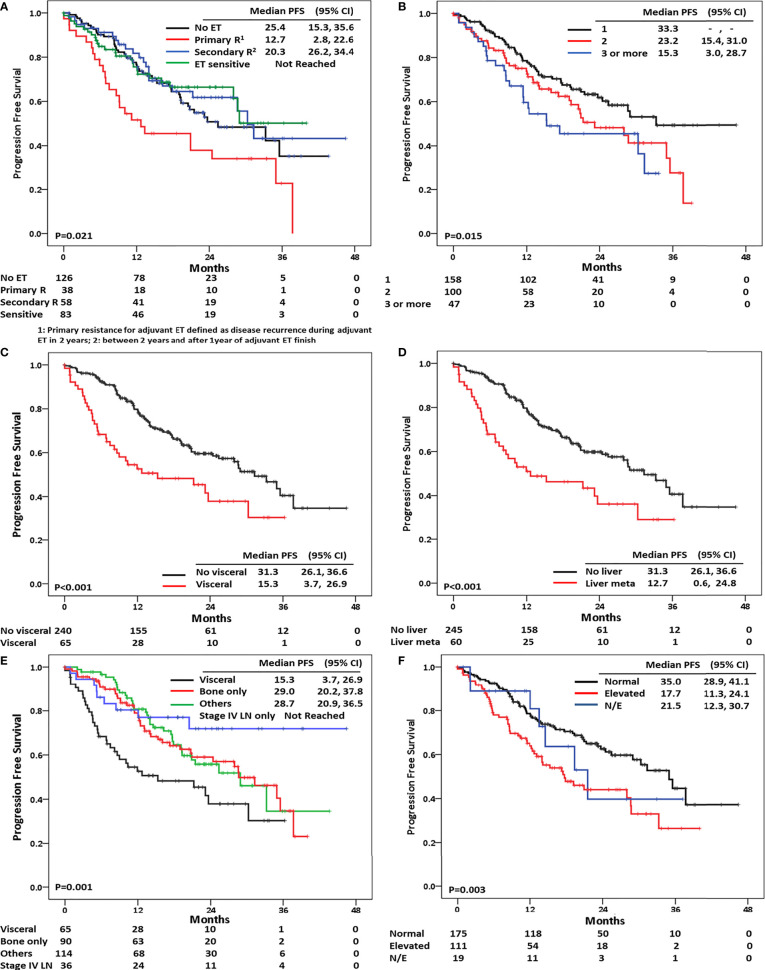
Progression-free survival according to **(A)** response to adjuvant endocrine treatment, **(B)** number of metastatic sites, **(C)** visceral metastasis, **(D)** liver metastasis, **(E)** metastatic sites, and **(F)** baseline CA-15-3 level.

In terms of endocrine resistance to adjuvant ET, four categories were made: primary resistance, which was defined as disease recurrence after less than 2 years of adjuvant ET; secondary resistance, defined as disease recurrence either 2 years or more after adjuvant ET or less than 1 year after the completion of adjuvant ET; no endocrine resistance to adjuvant ET; and endocrine-naïve regardless of *de novo* or recurrent BC. In this analysis, patients with primary resistance to ET had the worst PFS, and the median PFS of these patients was 12.7 months (*p* = 0.021) (**Figure 3A**). Patients having three or more metastatic sites also had worse prognoses compared to those with one or two metastatic sites (median PFS of one *vs.* two *vs.* three or more metastatic sites: 33.3 *vs.* 23.2 *vs.* 15.3 months; *p* = 0.015) (**Figure 3B**).

In 65 patients with visceral metastasis, we observed 60 patients with liver metastasis and nine patients with brain metastasis. Visceral metastasis was associated with poor PFS compared to non-visceral metastasis (median PFS of visceral *vs.* non-visceral metastasis: 15.3 *vs.* 31.3 months; *p* < 0.001) (**Figure 3C**), and liver metastasis was associated with poor prognosis compared with any other metastatic sites (median PFS of liver metastasis *vs.* others: 12.7 *vs.* 31.3 months; *p* < 0.001) (**Figure 3D**). With regards to metastatic sites, those with lymph node or skin metastases had the longest PFS compared with those having other metastatic lesions (median PFS of lymph node or skin metastases: not reached; *p* = 0.001). In addition, bone-only disease had a superior survival outcome compared to BC with visceral metastasis (median PFS of bone-only disease *vs.* others *vs.* visceral metastasis: 29.0 *vs.* 28.7 *vs.* 15.3; *p* = 0.001) (**Figure 3E**).

The elevation of baseline serum tumor markers CA-15-3 and CEA was also associated with short PFS duration (*p* = 0.003 and *p* = 0.004, respectively) (**Figure 3F** and **Supplementary Figure S3**).

### Multivariate Analysis of the Factors Affecting PFS Following Palbociclib With Letrozole

**Figure 4:**
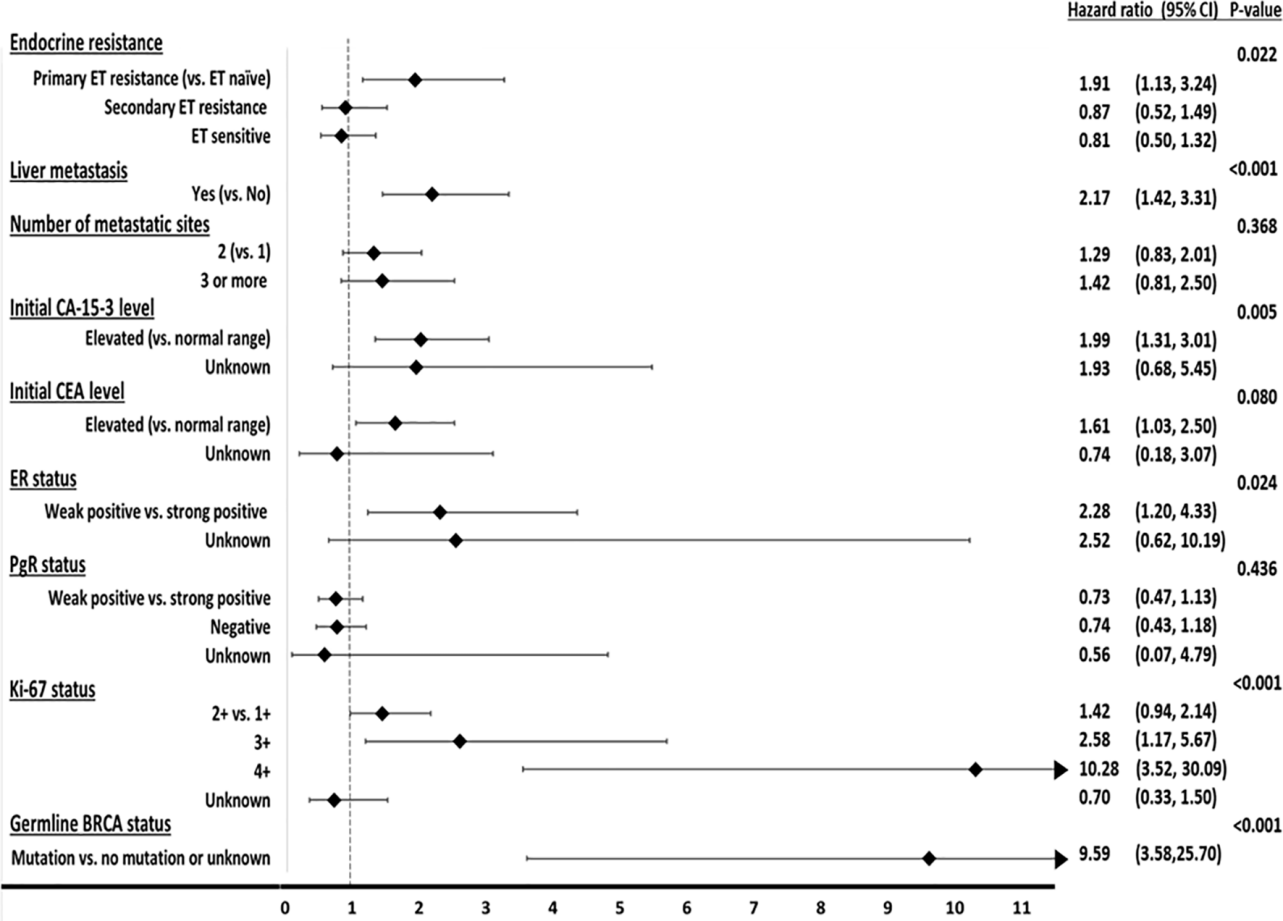
Forest plot of multivariate analysis for factors affecting progression-free survival.

Multivariate analysis was performed using the characteristics affecting PFS following palbociclib in combination with letrozole for the treatment of HR+, HER2-MBC. We excluded visceral metastasis from this analysis because this factor overlapped with liver metastasis. In this analysis, primary resistance to adjuvant ET (hazard ratio: 1.91, 95% CI: 1.13, 3.24; *p* = 0.022), presence of liver metastasis (hazard ratio: 2.17, 95% CI: 1.42, 3.31; *p* < 0.001), initial elevation of serum CA-15-3 level (hazard ratio: 1.99, 95% CI: 1.31, 3.01; *p* = 0.005), weak ER positivity (hazard ratio: 2.28, 95% CI: 1.20, 4.33; *p* = 0.024), Ki-67 3+ or 4+ [hazard ratios: 2.58 (95% CI: 1.17, 5.67) and 10.28 (95% CI: 3.52, 30.09); *p* < 0.001], and presence of BRCA2 mutation (hazard ratio: 9.59, 95% CI: 3.58, 25.70; *p* < 0.001) were associated with short PFS (**Figure 4** and **Supplementary Table S2**).

We excluded the unknown values of six factors and did a binary division of these factors for prediction model development. Therefore, we performed a further multivariate analysis with the values from 256 patients. In this analysis, these six factors consistently had an effect on PFS with statistical significance. The hazard ratio of primary resistance to adjuvant ET for short PFS was 2.27 (95% CI: 1.39, 3.72; *p* = 0.001), that for the presence of liver metastasis was 2.10 (95% CI: 1.35, 3.25; *p* = 0.001), that for the initial elevation of CA-15-3 was 2.26 (95% CI: 1.51, 3.40; *p* < 0.001), that for weak expression of ER status was 2.20 (95% CI: 1.10, 4.41; *p* = 0.027), that for high Ki-67 expression (3+ and 4+) was 3.42 (95% CI: 1.87, 6.56; *p* < 0.001), and that for BRCA2 mutation was 8.54 (95% CI: 2.57, 28.36; *p* < 0.001) (**Table 2**).

**Table 2:** Multivariate analysis using binary variables for progression-free survival following palbociclib with letrozole (*N* = 256).

Characteristics	*N* (%)	Hazard ratio	95% confidence interval		*p*-value
Resistance to adjuvant ET					0.001
Other	221 (86.3)	Ref			
Primary ET resistance	35 (13.7)	2.27	1.39	3.72	
Liver metastasis					0.001
No	203 (79.3)	Ref			
Yes	53 (20.7)	2.10	1.35	3.25	
Initial elevation of CA-15-3					<0.001
Normal range	156 (60.9)	Ref			
Elevated	100 (39.1)	2.26	1.51	3.40	
Estrogen receptor status					0.027
Strong positivity	242 (94.5)	Ref			
Weak positivity	14 (5.5)	2.20	1.10	4.41	
Ki-67					<0.001
1+, 2+	239 (93.4)	Ref			
3+, 4+	17 (6.6)	3.42	1.87	6.56	
BRCA mutation					<0.001
No mutation or unknown	253 (98.8)	Ref			
BRCA2 mutation	3 (1.2)	8.54	2.57	28.36	

ET, endocrine therapy.

### Prediction Model for Primary Resistance to Palbociclib With Letrozole

A prediction model for primary resistance to palbociclib with letrozole as the first-line treatment was developed considering patients with HR+, HER2-MBC. We used the values from 256 patients, and 33 events of disease progression in six months were observed.

Firth logistic regression for primary resistance suggested that the five factors—primary resistance to adjuvant ET (odds ratio, OR: 1.14, 95% CI: 0.06, 2.18; *p* = 0.038), liver metastasis (OR: 1.56, 95% CI: 0.71, 2.42; *p* < 0.001), initial elevation of CA-15-3 (OR: 1.51, 95% CI: 0.63, 2.49; *p* < 0.001), weak expression of ER (OR: 2.22, 95% CI: 0.99, 3.51; *p* < 0.001), and BRCA mutation (OR: 2.85, 95% CI: 0.75, 5.31; *p* = 0.010)—affected the 6-month PFS (**Table 3**).

**Table 3:** Predictive model for primary resistance to palbociclib with letrozole as first-line treatment for HR+, HER2-advanced breast cancer using firth logistic regression (*N* = 256).

Characteristics	*N* (%)	Odds ratio	95% confidence interval		*p*-value
Disease progression	33 (12.9)				
Primary resistance to adjuvant ET^a^		1.14	0.06	2.18	0.038
Presence of liver metastasis		1.56	0.71	2.42	<0.001
Initial elevation of CA-15-3		1.51	0.63	2.49	<0.001
Estrogen receptor weak positivity^b^		2.22	0.99	3.51	<0.001
Ki-67 3+ or 4+		0.99	0.62	2.24	0.167
Presence of BRCA mutation		2.85	0.75	5.31	0.010

a Endocrine therapy.

b Weak or unknown positivity.

**Figure 5:**
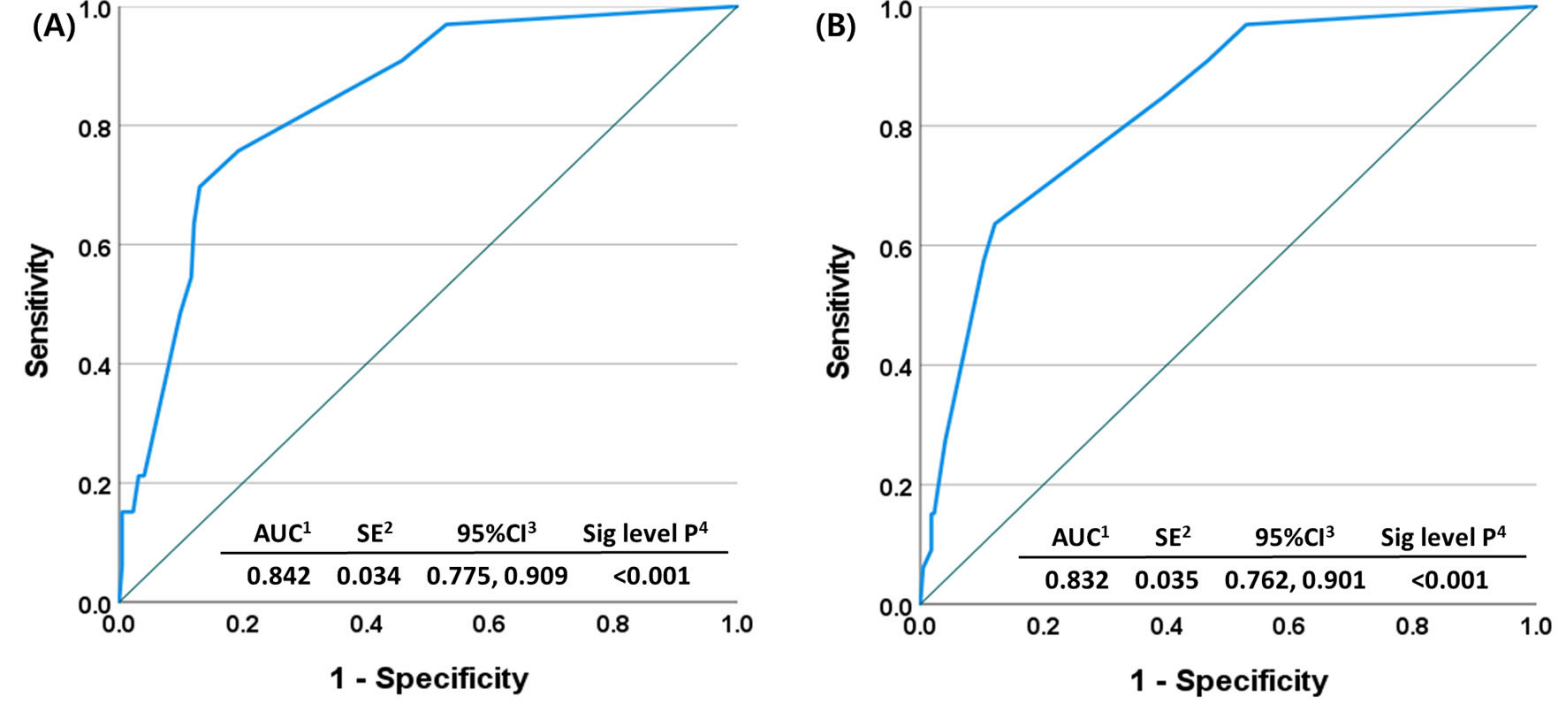
Receiver operating characteristic curve for primary resistance to palbociclib with letrozole as the first-line treatment for HR+ HER2- MBC **(A)** original set (*n* = 256) and **(B)** validation set (*n* = 182).

Then, we developed a prediction model for the primary resistance to palbociclib with letrozole. In this analysis, the prediction model with five risk factors had 0.842 in AUC of the ROC curve (95% CI: 0.775, 0.909; *p* < 0.001), and the overall model quality was 0.78 (**Figure 5A** and **Supplementary Figure S4A**). Internal validation with bootstrap resampling datasets was performed (*n* = 182). In the validation set, AUC was 0.832 (95% CI: 0.762, 0.901; *p* < 0.001), and the overall model quality was 0.76 (**Supplementary Table S3**, **Figure 5B**, and **Supplementary Figure S4B**).

### Primary Resistance Model With the Five Risk Factors

We analyzed the risk of primary resistance of palbociclib with letrozole according to the five risk factors. First, we performed a survival analysis according to the number of risk factors.

In the survival analysis, the median PFS of patients with no risk factor was not reached, and it was 28.0-month PFS in patients with one risk factor, 8.2-months PFS in patients with two risk factors, and 6.8-month PFS in patients who had three risk factors (*p* < 0.001) (**Supplementary Figure S5A**). The primary resistance of palbociclib with letrozole model also suggested that the risk of primary resistance increased as more risk factors existed [OR of one risk factor *vs.* no risk factor (ref): 2.18, 95% CI: 0.72, 4.41, *p* = 0.002; OR of two risk factors: 4.01, 95% CI:2.57, 6.25, *p* < 0.001; OR of three risk factors: 4.00, 95% CI: 1.99, 6.50, *p* < 0.001] (**Supplementary Table S4**), and AUC was 0.830 (95% CI: 0.761, 0.898) (*p* < 0.001) (**Supplementary Figure S5B**).

**Figure 6:**
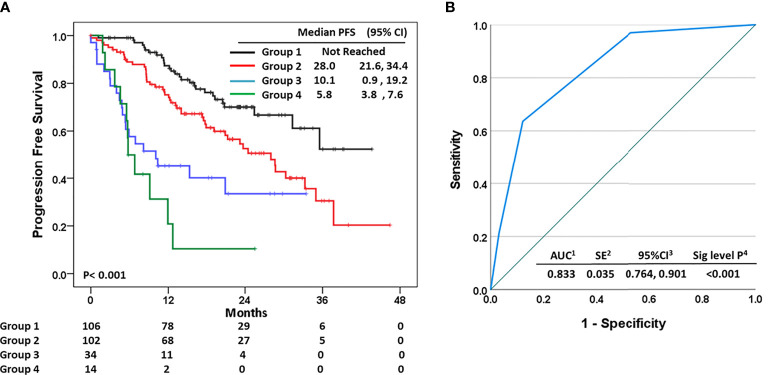
**(A)** Progression-free survival according to the risk groups of the prediction model. **(B)** Receiver operating characteristic curve for primary resistance to palbociclib with letrozole as the first-line treatment for HR+ HER2- MBC regarding the risk groups.

We also developed a primary resistance model according to the value made of the previous Firth logistic regression with five risk factors. We divided these into four groups: group 1 did not have any risk factors, group 2 had one risk factor except BRCA mutation, group 3 had two risk factors except weak ER positivity, and group 4 consisted of BRCA mutation, three risk factors, and two risk factors including weak ER positivity (**Supplementary Table S5**). The median PFS of group 1 was not reached, and it was 28.0-month PFS in group 2, 10.1-month PFS in group 3, and 5.8-month PFS in group 4 (*p* < 0.001) (**Figure 6A**). The prediction model also precisely expected the primary resistance of palbociclib with letrozole [OR of group 2 *vs.* 1 (ref): 2.18, 95% CI: 0.72, 4.41, *p* = 0.002; OR of group 3: 3.91, 95% CI: 2.42, 6.16, *p* < 0.001; OR of group 3: 4.25, 95% CI: 2.56, 6.60, *p* < 0.001, and AUC of 0.830 (95% CI: 0.761, 0.898, *p* < 0.001)] (**Table 4** and **Figure 6B**).

**Table 4:** Predictive model for primary resistance to palbociclib with letrozole as first-line treatment for HR+, HER2-advanced breast cancer according to risk factor characteristics (*N* = 256).

Characteristics	*N* (%)	Odds ratio	95% confidence interval		*p*-value
Disease progression	33 (12.9)				<0.001
No risk factor (group 1)		Ref	Ref	Ref	Ref
One risk factor (group 2)^a^		2.18	0.72	4.41	0.002
Group 3^b^		3.91	2.42	6.16	<0.001
Group 4^c^		4.25	2.56	6.60	<0.001

a Except BRCA mutation.

b Two risk factors except weak estrogen receptor (ER) positivity and BRCA mutation.

c BRCA mutation regardless of the number of risk factors, three risk factors, and two risk factors including weak ER positivity.

### Second-Line Treatment After Progression of the First-Line Palbociclib With Letrozole

Of 38 patients with primary resistance to palbociclib in combination with letrozole, 35 patients received second-line treatment. Capecitabine was the most commonly used therapeutic regimen, followed by taxane, everolimus in combination with exemestane, fulvestrant, and others. The overall 6-month disease control rate was 31.4% (31.3% for capecitabine, 42.9% for taxane, 40.0% for everolimus, and 50.0% for fulvestrant) (**Table 5**).

**Table 5:** Second-line treatment after progression from endocrine therapy with palbociclib and letrozole (*N* = 38).

Regimen	*N* (%)	3-month DCR (%)	6-month DCR (%)	12-month DCR (%)
Capecitabine	16 (43.2)	10 (62.5)	5 (31.3)	2 (12.5)
Taxane-based chemotherapy	7 (24.3)	5 (71.4)	3 (42.9)	1 (14.3)
Everolimus with exemestane	5 (13.5)	4 (80.0)	2 (40.0)	1 (20.0)
Fulvestrant	2 (5.4)	1 (50.0)	1 (50.0)	1 (50.0)
Clinical trial	5 (13.5)	2 (40.0)	0 (0.0)	0 (0.0)
No further treatment	3 (7.9)	–	–	–
Total	38 (100.0)	22 (62.9)	11 (31.4)	5 (14.3)

DCR, disease control rate.

## Discussion

This study considered the real-world data of palbociclib with letrozole as the first-line treatment for HR+, HER2-MBC. The median PFS was 28.7 months, and primary resistance to adjuvant ET, liver metastasis, initial elevation of CA-15-3 level, weak ER expression, high expression of Ki-67, and BRCA2 mutation were associated with poor PFS. Our prediction model suggested that these six parameters affected the primary resistance to palbociclib with letrozole, and this model had an AUC of 0.844.

ET is the first-line treatment for HR+, HER2-MBC even in the presence of visceral disease, unless there is visceral crisis (3, 4). In the era of CDK4/6 inhibitor, CDK4/6 inhibitors combined with ET is the standard treatment strategy for HR+, HER2-MBC as the first or second line (3). However, resistance to this combination therapy inevitably develops, and some patients do not benefit from CDK4/6 inhibitor with AI treatment (8–10).

In our study, germline BRCA mutation was the most powerful predictive marker for primary resistance to palbociclib with letrozole. Of five patients harboring germline BRCA2 mutation, the median PFS was just 5.7 months, and the hazard ratio for primary resistance in those with BRCA2 mutation was 22.57. However, the number of patients with BRCA mutations in this study was small, and there are currently no other data supporting this association. Further preclinical and clinical research is warranted.

The initial elevation of serum CA-15-3 was also strongly associated with primary resistance to palbociclib with letrozole. The current European Society for Medical Oncology and American Society of Clinical Oncology guideline suggests that CA-15-3 and CEA are not recommended for BC screening, diagnosis, staging, or recurrence evaluation (26, 27). The clinical values of these tumor markers are not well established but might be an aid to evaluate and monitor the response to treatment, particularly in patients with non-measurable metastatic disease with elevation of these markers (3). Many investigators have tried to reveal the clinical values of serum tumor markers, and recent research has suggested CA-15-3 elevation at the time of initial metastasis in 37% of patients with MBC, and 62% of patients had an increase in CA-15-3 level at the diagnosis of metastasis (28). Another study also suggested that the CEA and CA-15-3 levels were useful to detect metastasis early, and their elevations were associated with unfavorable clinicopathological parameters (29).

In our study, CA-15-3 serum level was associated with primary resistance to palbociclib with letrozole after adjustment through a multivariate analysis. We suggest CA-15-3 to be an independent predictive marker for primary resistance to palbociclib with letrozole, not a secondary finding associated with clinicopathologic parameters. Moreover, serum CA-15-3 testing had advantages with respect to convenience, easy accessibility, and low cost.

Liver metastasis was already suggested to associate with poor prognosis following CDK4/6 treatment in previous studies (30, 31). Our study also suggested that liver metastasis was associated with poor response to palbociclib with letrozole. Even considering visceral metastasis, including liver metastasis, CDK4/6 inhibition with AI was an effective treatment strategy compared with AI alone, according to previous clinical trials (8–10). However, some patients with visceral metastasis did not benefit from CDK4/6 inhibitors with ET, and cytotoxic chemotherapy would be better than ET for these patients; predictive biomarkers are urgently needed.

ER status was also associated with primary resistance to the first line of palbociclib treatment in combination with letrozole. Even though there was a small number of patients with weak ER+ MBC in this study, they had significantly worse PFS outcomes compared to patients with strong ER+ MBC. Recent guidelines described low ER tumors as having unique molecular features and, therefore, a distinct therapeutic response to endocrine therapy compared with high ER+ BC (32). In this study, ER status was divided into two groups according to Allred score (7 to 8 *vs.* 3–6). In terms of ER status, no previous studies, including clinical trials, have evaluated the association between ER expression level and the efficacy of CDK 4/6 inhibitors with ET. The results of this study suggested that the expression level of ER should be considered for the treatment of HR+, HER2-MBC with CDK4/6 inhibitors.

Previous research has suggested PgR and Ki-67 state to affect the efficacy of palbociclib (33). Our study also suggested that Ki-67 expression, but not PgR status, was related to PFS following a palbociclib-containing treatment.

Primary resistance to adjuvant ET was associated with primary resistance to the first-line treatment consisting of palbociclib with letrozole for HR+, HER2-MBC. Disease recurrence within 2 years of adjuvant ET was associated with primary resistance to palbociclib, but secondary resistance to ET was not.

Lastly, we developed a prediction model of primary resistance of palbociclib with letrozole as the first-line treatment for metastatic HR+, HER2-MBC. This model suggested that the number and the characteristics of risk factors easily predicted primary resistance and the PFS of a patient. Therefore, this model would be helpful to predict the response of patients to palbociclib with letrozole as the initial treatment.

Our study was a retrospective data analysis with data from about 300 patients in our registry. Therefore, we only performed internal validation to validate our primary resistance model. However, the result of internal validation was similar to that of the original data set with high AUC, and therefore we might suggest that our model was reliable.

Even though the current treatment guidelines recommended CDK 4/6 inhibitors in combination with ET as the first-line treatment in HR+, HER2-MBC, cytotoxic chemotherapy would be more beneficial compared to the use of palbociclib with letrozole in some patients with poor clinico-pathologic parameters. Among 38 patients who underwent disease progression after the first-line palbociclib with letrozole within 6 months, 67.5% of patients had been treated with cytotoxic chemotherapy, and 34.8% of these patients had more than 6 months of PFS following a second-line cytotoxic treatment. Therefore, our prediction model suggested that these clinico-pathologic parameters would be helpful for deciding the first-line treatment in a subset of HR+, HER2-MBC patients. It is necessary to note that these findings are just hypothesis-generating, especially considering that no predictive biomarkers have yet been established related to treatment consisting of CDK 4/6 inhibitors with ET.

In conclusion, we explored palbociclib in combination with letrozole as the first-line treatment for HR+, HER2-MBC and developed a prediction model for primary resistance to the first-line treatment of palbociclib with letrozole. Our prediction model might be helpful for considering the first-line treatment strategies in HR+, HER2-MBC. Further well-designed clinical trials are warranted to validate our prediction model.

## Data Availability Statement

The original contributions presented in the study are included in the article/**Supplementary Material**. Further inquiries can be directed to the corresponding author.

## Ethics Statement

This study was reviewed and approved by the Institutional Review Board of Samsung Medical Center, Seoul, South Korea (IRB no. 2021-07-131). This study was performed in accordance with the Declaration of Helsinki and Good Clinical Practice guidelines. The requirement for informed consent was waived due to the use of de-identified medical records with clinical data. Written informed consent for participation was not required for this study in accordance with the national legislation and the institutional requirements.

## Author Contributions

J-YK conceived and planned the experiments. J-YK and JO carried out the analyses and experiments. J-YK, YP, JA, and Y-HI contributed to the collection of samples and clinical data. J-YK and Y-HI contributed to the interpretation of the results and took the lead in writing the manuscript. Y-HI supervised the project. All authors contributed to the article and approved the submitted version.

## Funding

This work was supported by an Institute for Information and Communications Technology Promotion grant funded by the Korean government (2018-0-00861, Intelligent SW Technology Development for Medical Data Analysis), a grant from the Korea Health Technology R and D Project through the Korea Health Industry Development Institute funded by the Ministry of Health and Welfare, Republic of Korea (grant number: HR20C0025), and grants from the National Research Foundation of Korea (NRF-2017R1D1A1B03028446 and NRF-2020R1F1A1072616).

## Conflict of Interest

The authors declare that the research was conducted in the absence of any commercial or financial relationships that could be construed as a potential conflict of interest.

## Publisher’s Note

All claims expressed in this article are solely those of the authors and do not necessarily represent those of their affiliated organizations, or those of the publisher, the editors and the reviewers. Any product that may be evaluated in this article, or claim that may be made by its manufacturer, is not guaranteed or endorsed by the publisher.

## Supplementary Material

Supplementary Figure 1 **(A)** Progression-free survival and **(B)** overall survival.

Supplementary Figure 2 Progression-free survival according to germline BRCA mutation, BRCA wild type, and BRCA unknown.

Supplementary Figure 3 Progression-free survival according to baseline CEA level.

Supplementary Figure 4 Overall model quality of receiver operating characteristic curve for primary resistance to palbociclib with letrozole as the first-line treatment for HR+ HER2- MBC **(A)** original set (*n* = 256) and **(B)** validation set (*n* = 182).

Supplementary Figure 5 **(A)** Progression-free survival according to the number of risk factors. **(B)** Receiver operating characteristic curve for primary resistance to palbociclib with letrozole as the first-line treatment for HR+ HER2- metastatic breast cancer regarding the number of risk factor.
