# Supplementary figures and images for: Which Clinicopathologic Parameters Suggest Primary Resistance to Palbociclib in Combination With Letrozole as the First-Line Treatment for Hormone Receptor-Positive, HER2-Negative Advanced Breast Cancer?
Source: Front Oncol. 2021 Oct 21;11:759150. doi: 10.3389/fonc.2021.759150 (PMC8566811; doi:10.3389/fonc.2021.759150)

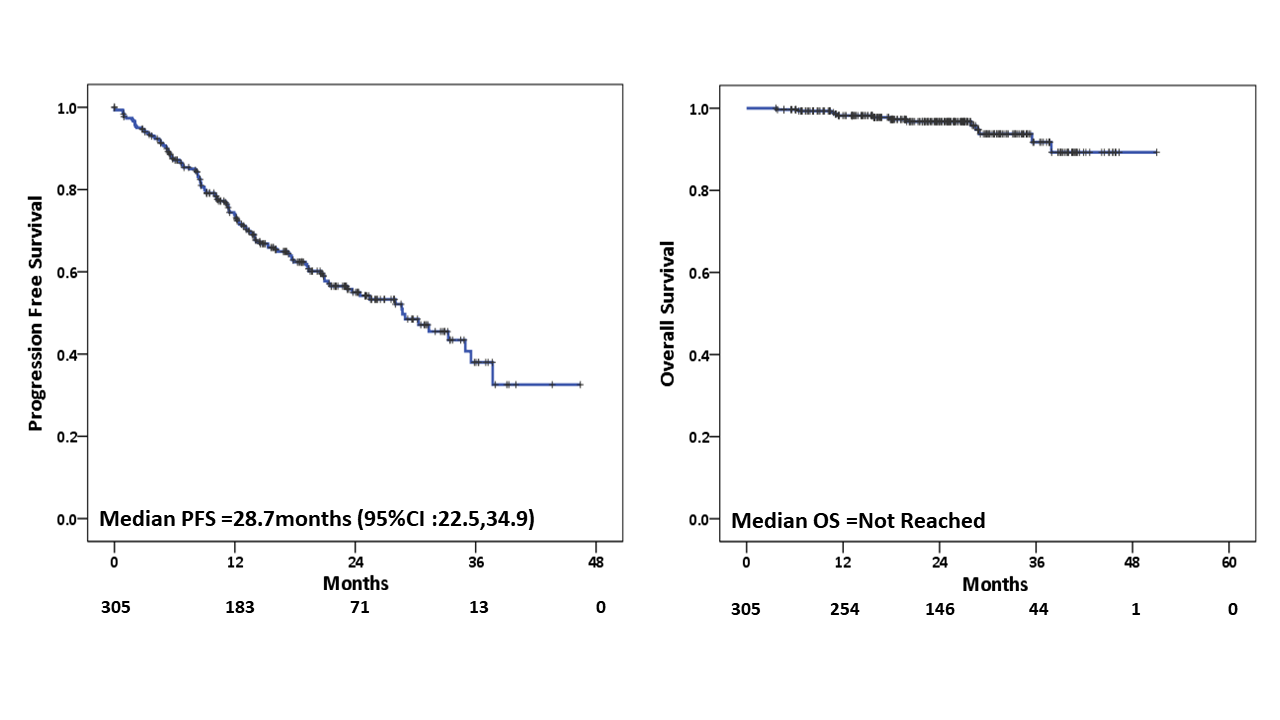

Supplement: Supplementary Figure 1 — (A) Progression-free survival and (B) overall survival. [file Image_1.tif]

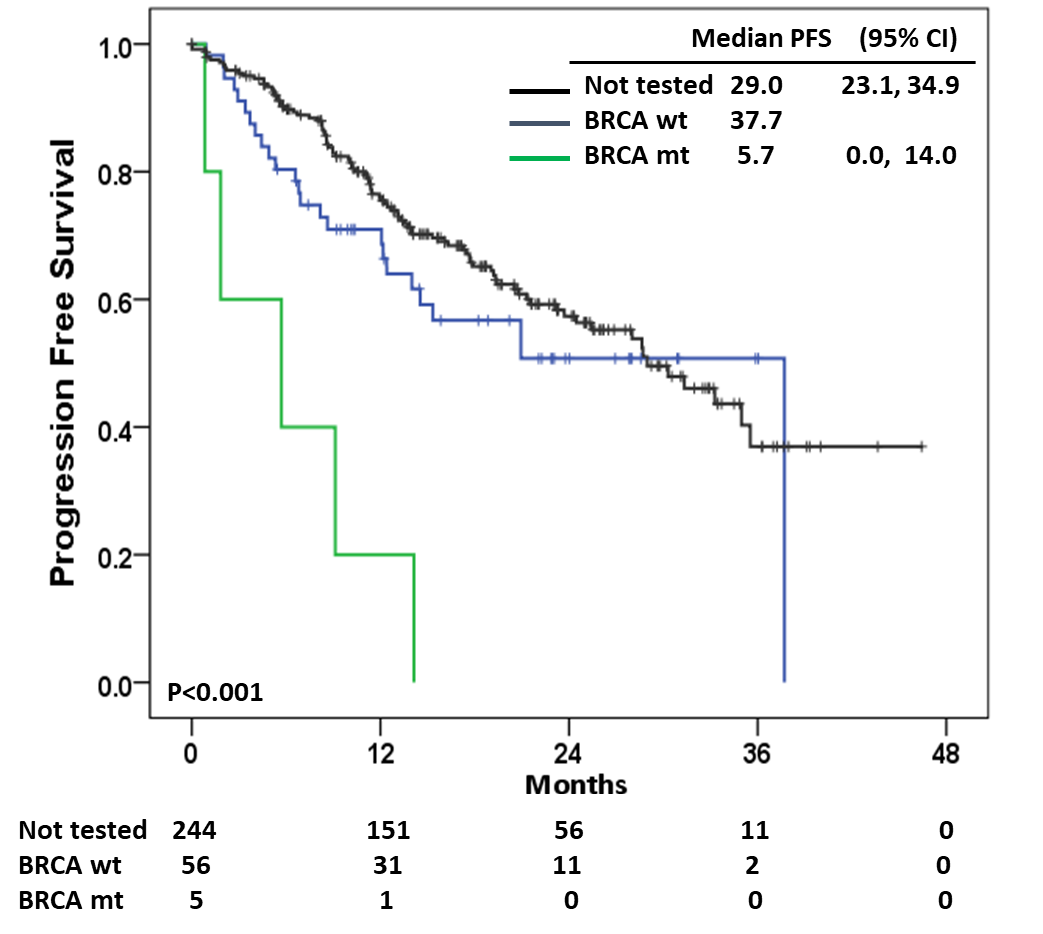

Supplement: Supplementary Figure 2 — Progression-free survival according to germline BRCA mutation, BRCA wild type, and BRCA unknown. [file Image_2.tif]

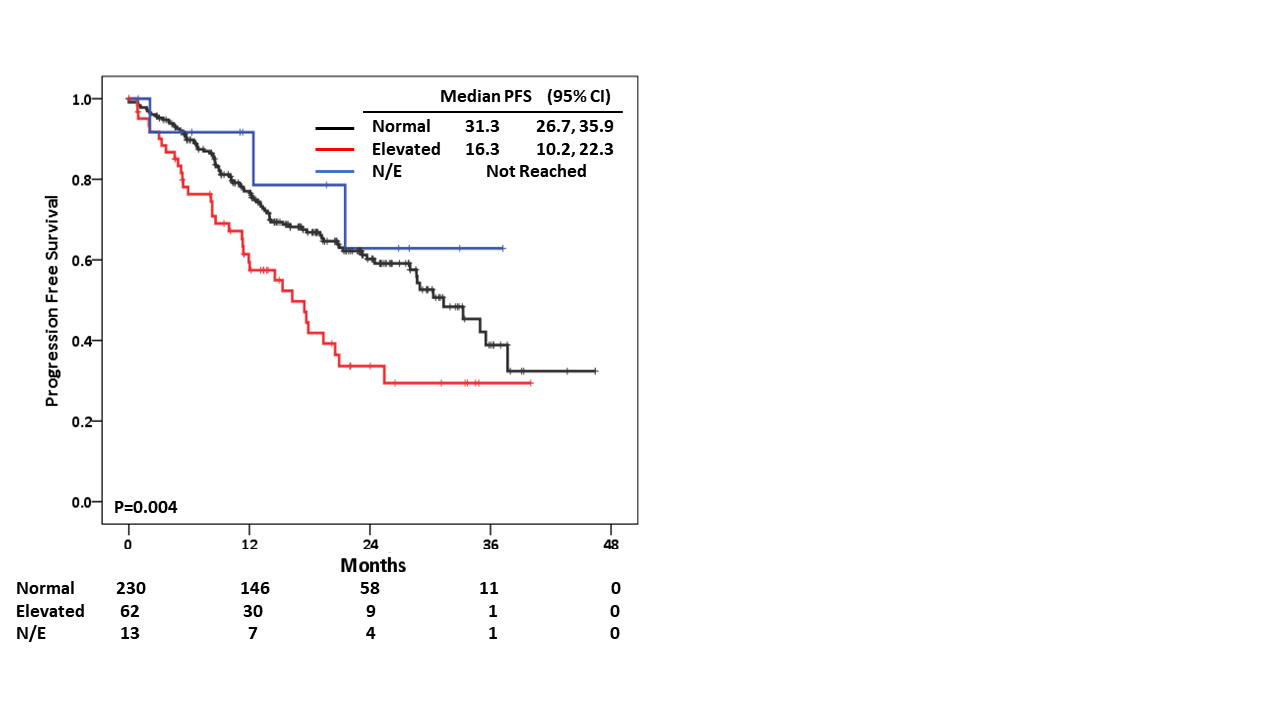

Supplement: Supplementary Figure 3 — Progression-free survival according to baseline CEA level. [file Image_3.tif]

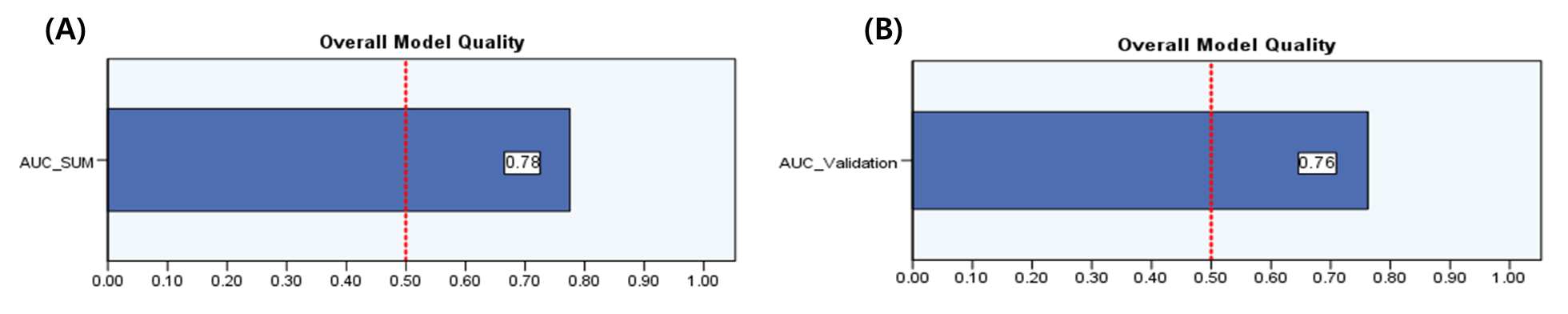

Supplement: Supplementary Figure 4 — Overall model quality of receiver operating characteristic curve for primary resistance to palbociclib with letrozole as the first-line treatment for HR+ HER2- MBC (A) original set (n = 256) and (B) validation set (n = 182). [file Image_4.tif]

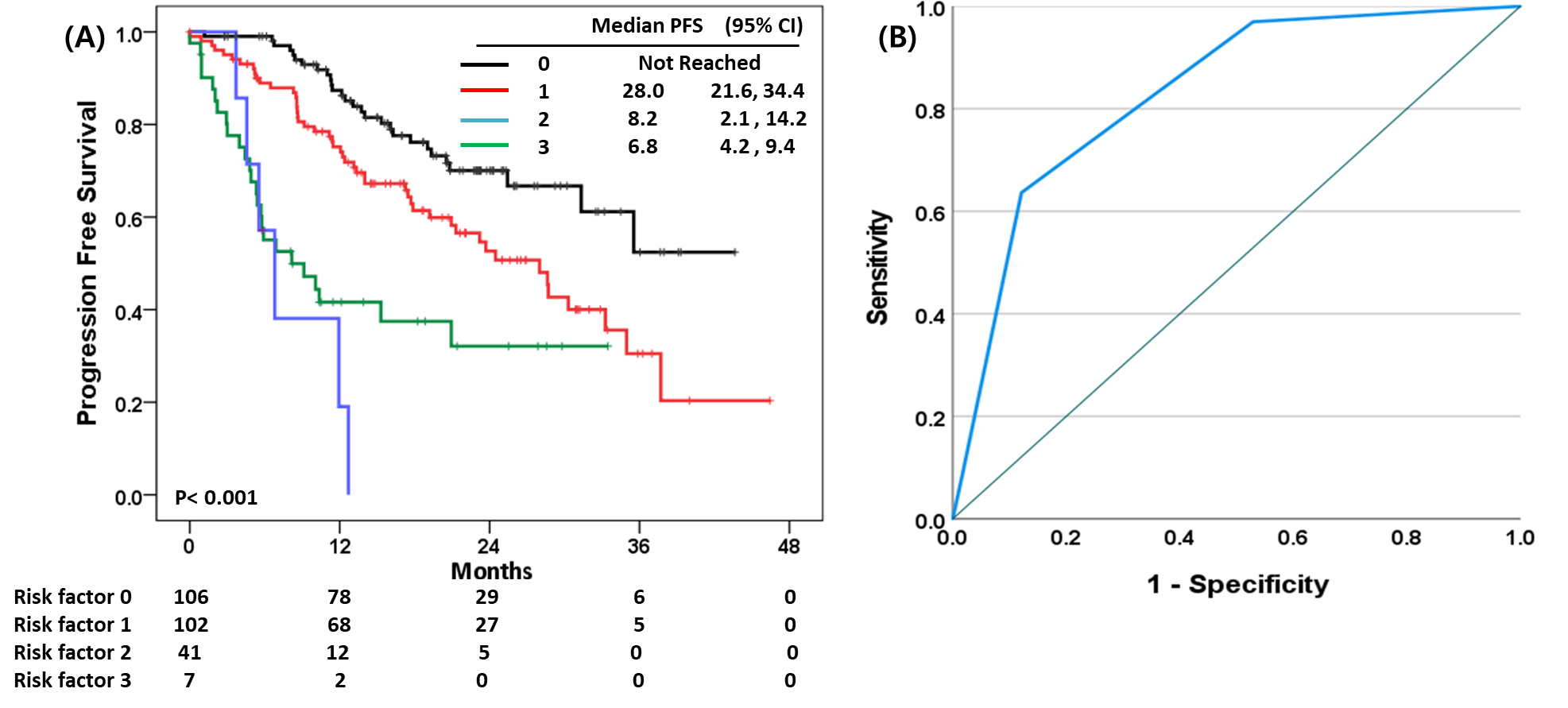

Supplement: Supplementary Figure 5 — (A) Progression-free survival according to the number of risk factors. (B) Receiver operating characteristic curve for primary resistance to palbociclib with letrozole as the first-line treatment for HR+ HER2- metastatic breast cancer regarding the number of risk factor. [file Image_5.tif]
