# Supplementary material for: Which Clinicopathologic Parameters Suggest Primary Resistance to Palbociclib in Combination With Letrozole as the First-Line Treatment for Hormone Receptor-Positive, HER2-Negative Advanced Breast Cancer?
Source: Front Oncol. 2021 Oct 21;11:759150. doi: 10.3389/fonc.2021.759150 (PMC8566811; doi:10.3389/fonc.2021.759150)
Supplement: Supplementary file 7 [file DataSheet_2.docx]

**Supplementary Table 2. Multivariate analysis of progression-free survival following first-line palbociclib with letrozole (N=305)**

| **Characteristics** |  | **N (%)** | **Hazard ratio** | **95% Confidence interval** | | **p-value** |
| --- | --- | --- | --- | --- | --- | --- |
| **Endocrine resistance** | **ET^1^-naïve** | **126 (41.3)** | **Ref** |  |  | **0.022** |
|  | **Primary resistance to adjuvant ET** | **38 (12.5)** | **1.91** | **1.13** | **3.24** |  |
|  | **Secondary resistance to adjuvant ET** | **58 (19.0)** | **0.87** | **0.52** | **1.49** |  |
|  | **ET-sensitive** | **83 (27.2)** | **0.81** | **0.50** | **1.32** |  |
| **Liver metastasis** | **No** | **245 (80.3)** | **Ref** |  |  | **<0.001** |
|  | **Yes** | **60 (19.7)** | **2.17** | **1.42** | **3.31** |  |
| **Number of metastatic sites** | **1** | **158 (51.8)** | **Ref** |  |  | **0.368** |
|  | **2** | **100 (32.8)** | **1.29** | **0.83** | **2.01** |  |
|  | **3 or more** | **47 (15.4)** | **1.42** | **0.81** | **2.50** |  |
| **Initial CA-15-3** | **Normal range** | **175 (57.4)** | **Ref** |  |  | **0.005** |
|  | **Elevated** | **111 (36.4)** | **1.99** | **1.31** | **3.01** |  |
|  | **Unknown** | **19 (6.2)** | **1.93** | **0.68** | **5.45** |  |
| **Initial CEA** | **Normal range** | **230 (75.4)** | **Ref** |  |  | **0.080** |
|  | **Elevated** | **62 (20.3)** | **1.61** | **1.03** | **2.50** |  |
|  | **Unknown** | **13 (4.3)** | **0.74** | **0.18** | **3.07** |  |
| **Estrogen receptor** | **Strong positivity (Allred score 7-8)** | **278 (91.1)** | **Ref** |  |  | **0.024** |
|  | **Weak positivity (Allred score 3-6)** | **18 (5.9)** | **2.28** | **1.20** | **4.33** |  |
|  | **Unknown** | **9 (3.0)** | **2.52** | **0.62** | **10.19** |  |
| **Progesterone receptor** | **Strong positivity (Allred score 7-8)** | **147 (48.2)** | **Ref** |  |  | **0.436** |
|  | **Weak positivity (Allred score 3-6)** | **74 (24.3)** | **0.73** | **0.47** | **1.13** |  |
|  | **Negative** | **75 (24.6)** | **0.74** | **0.43** | **1.18** |  |
|  | **Unknown** | **9 (3.0)** | **0.56** | **0.07** | **4.79** |  |
| **Ki-67** | **1+** | **167 (54.8)** | **Ref** |  |  | **<0.001** |
|  | **2+** | **83 (27.2)** | **1.42** | **0.94** | **2.14** |  |
|  | **3+** | **14 (4.6)** | **2.58** | **1.17** | **5.67** |  |
|  | **4+** | **4 (1.3)** | **10.28** | **3.52** | **30.09** |  |
|  | **Unknown** | **37 (12.1)** | **0.70** | **0.33** | **1.50** |  |
| **BRCA mutation** | **No mutation or unknown** | **300 (98.4)** | **Ref** |  |  | **<0.001** |
|  | **BRCA mutation** | **5 (1.6)** | **9.59** | **3.58** | **25.70** |  |

**1: Endocrine therapy**

**Supplementary Table 3. Internal validation with bootstrap resampling datasets (N=182)**

| **Characteristics** | **N (%)** | **Odds ratio** | **95% confidence interval** | | **p-value** |
| --- | --- | --- | --- | --- | --- |
| **Disease progression** | **25 (13.7)** |  |  | |  |
| **Primary resistance to adjuvant ET^1^** |  | **1.82** | **0.55** | **3.14** | **0.005** |
| **Presence of liver metastasis** |  | **1.22** | **0.12** | **2.32** | **0.030** |
| **Initial elevation of CA-15-3** |  | **1.95** | **0.84** | **3.25** | **<0.001** |
| **Estrogen receptor weak positivity^2^** |  | **1.95** | **0.20** | **3.67** | **0.030** |
| **Ki-67 3+ or 4+** |  | **0.73** | **-1.59** | **2.42** | **0.472** |
| **Presence of BRCA mutation** |  | **2.63** | **0.52** | **5.09** | **0.016** |

**1: Endocrine therapy; 2: Weak or unknown positivity**

**Supplementary Table 4. Predictive model for primary resistance to palbociclib with letrozole as first-line treatment for HR+, HER2- advanced BC according to number of risk factors (N=256)**

| **Characteristics** | **N (%)** | **Odds ratio** | **95% confidence interval** | | **p-value** |
| --- | --- | --- | --- | --- | --- |
| **Disease progression** | **33 (12.9)** |  |  | | **<0.001** |
| **0** |  | **ref** | **ref** | **ref** | **ref** |
| **1** |  | **2.18** | **0.72** | **4.41** | **0.002** |
| **2** |  | **4.01** | **2.57** | **6.25** | **<0.001** |
| **3** |  | **4.00** | **1.99** | **6.50** | **<0.001** |
